# Waste and Greenhouse Gas Emissions Produced from Ophthalmic Surgeries: A Scoping Review

**DOI:** 10.3390/ijerph22010051

**Published:** 2024-12-31

**Authors:** Brian Morris, Jenna Tauber, Anvit Rai, Cassandra Thiel, Tiana J. Geringer, Umar K. Mian

**Affiliations:** 1 NYU Langone Health, New York, NY 10016, USA; brian.morris@nyulangone.org; 2 Manhattan Eye, Ear & Throat Hospital, New York, NY 11432, USA; jtauber@northwell.edu; 3 Stony Brook School of Medicine, New York, NY 11794, USA; anvit72@gmail.com; 4 Departments of Population Health and Ophthalmology, NYU Langone Health, New York, NY 10016, USA; cassandra.thiel@nyulangone.org; 5 Department of Ophthalmology & Visual Sciences, Montefiore Medical Center, Albert Einstein College of Medicine, New York, NY 10461, USA; ttoribio@montefiore.org

**Keywords:** ophthalmology, cataract, surgery, waste, emissions, greenhouse, carbon, LCA

## Abstract

(1) Background: Healthcare is a major contributor to global greenhouse gas (GHG) emissions, especially within the surgical suite. Ophthalmologists play a role, since they frequently perform high-volume procedures, such as cataract surgery. This review aims to summarize the current literature on surgical waste and GHG emissions in ophthalmology and proposes a framework to standardize future studies. (2) Methods: Protocol and reporting methods were based on PRISMA guidelines for scoping reviews. Articles that reported any quantitative measurement of waste or GHGs produced from ophthalmic surgeries were eligible for inclusion. Commentaries, opinion papers, reviews and articles in a non-English language were excluded. (3) Results: A total of 713 articles were reviewed, with 10 articles found to meet inclusion criteria. Six studies produced level 3 evidence, two level 4 evidence, and one level 5 evidence. According to studies, most of the GHGs came from procurement of surgical materials, followed by travel emissions and building energy. (4) Conclusions: Research on waste and GHGs produced in ophthalmic surgery is limited, and existing studies utilize varied approaches to quantify this waste. We propose a standardized waste-lifecycle framework for researchers to organize future research. Such standardization will help in comparing studies and may uncover more opportunities to implement impactful waste reduction strategies in ophthalmology.

## 1. Introduction

Climate change is a significant public health issue [1]. Global warming has been linked to many conditions such as poor mental health, autoimmune diseases and vector-borne illnesses [2,3,4]. Ophthalmology is also affected, as increasing temperatures are linked to increased incidences of cataracts, fungal keratitis, and trachoma [5]. Despite this, healthcare delivery itself actively contributes to climate change, producing large amounts of greenhouse gases (GHGs) every year. To estimate the effect these GHGs will have, the global warming potential of each gas over a 100-year period relative to carbon dioxide (CO_2_), known as the GWP100, is used to provide a standardized unit of global warming potential for all GHGs [6]. For instance, the United States (US) healthcare system alone produces 479 million tons of CO_2_ each year, accounting for 8% of the total US GHGs [7]. As climate change progresses and populations continue to grow, the demand for healthcare will only increase, along with its GHG emissions. To minimize the adverse effects on global health, we need to re-evaluate how we utilize resources in healthcare.

The field of ophthalmologic surgery is an ideal target for reducing healthcare emissions. Surgical suites are the most resource-intensive areas of hospitals, and most ophthalmologic surgeries are high-volume procedures [8]. Cataract surgery is one of the most performed surgeries in the world, with an estimated 20 million extractions performed globally and 3.7 million in the US annually [9]. Phacoemulsification (phaco) is the most common type of cataract surgery, and is the standard of care in the Western world [10]. However, multiple small incision cataract surgery (MSICS) is a manual procedure that does not utilize a phacoemulsification machine and is generally used for more complicated cases [11]. Other surgical subspecialties, such as trabeculectomies and vitreoretinal surgery, should also be examined, since they differ in length of procedure, materials used, necessary operative machinery, and number of pre/post-operative visits. Differences in these variables will likely lead to different degrees of waste production among the ophthalmic surgical subspecialties.

Current hospital sustainability measures focus on recycling, which makes up a small proportion of surgical emissions [12]. Discussed in this review, the implementation of multi-use materials likely has a much greater effect on reducing waste production and GHG emissions [13]. This exemplifies the need for a greater understanding of where waste and GHGs are produced from ophthalmic surgeries. This will allow for a more effective approach to reduce healthcare emissions while maintaining safe, efficacious practices.

This review aims to summarize the current knowledge on ophthalmologic surgical waste generation and GHG emissions. As this is a relatively new research area, we must use similar vocabulary and methodologies. Hence, we also address the methods used to measure surgical waste GHGs, and provide recommendations to allow for greater standardization and comparability in future studies.

## 2. Materials and Methods

The protocol and reporting methods of this review were based on PRISMA guidelines for scoping reviews [14].

### 2.1. Eligibility Criteria

Studies were eligible for inclusion if they reported any quantitative measure of waste or GHGs produced from ophthalmic surgery or processes necessary for the surgery to occur. All subspecialties of ophthalmic surgery were included. Articles were excluded if they were a review, opinion paper, commentary, or did not report quantitative measures of waste or environmental impacts.

### 2.2. Information Sources and Search Strategy

The following search engines and databases were utilized to perform this review: PubMed, Embase and Google Scholar. A search string based on inclusion criteria was generated and then modified to meet the specific needs of each database. A list of search strings can be found in Supplementary Table S1. All resulting articles were reviewed individually in PubMed and Embase. In Google Scholar, only the first 100 articles by relevance were reviewed, due to the large number of search results (n = 19,300).

### 2.3. Search Strategy and Literature Review

Articles were reviewed for inclusion by two independent researchers (BM, AR). Articles were first screened based on title/abstract and were then put up for full text review. Following full text review, duplicate articles were excluded. The screening and sorting of articles was carried out in collections for PubMed, Clipboard for Embase and favorites for Google Scholar. All disagreements were settled by the Principal Investigator (UM).

### 2.4. Levels of Evidence

Levels of evidence, defined as a system used to rank studies based on the design’s reliability and quality, were assessed using the Cochrane guidelines [15]. Levels of evidence are reported in Table 1 with other study characteristics.

### 2.5. Data Extraction and Analysis

All data were manually extracted from articles by one reviewer (BM) and were input into Microsoft Excel. Tables were then generated by grouping relevant data. A limited analysis of means, ranges and standard deviations was conducted using Excel functions. In this review, carbon footprint is defined as the amount of greenhouse gas emissions produced by an ophthalmic surgery in a single eye. Emissions are reported in kilograms of carbon dioxide equivalents (kg CO_2_eq), which is a widely accepted standardized unit to measure climate-related impacts.

## 3. Results

**Figure 1:**
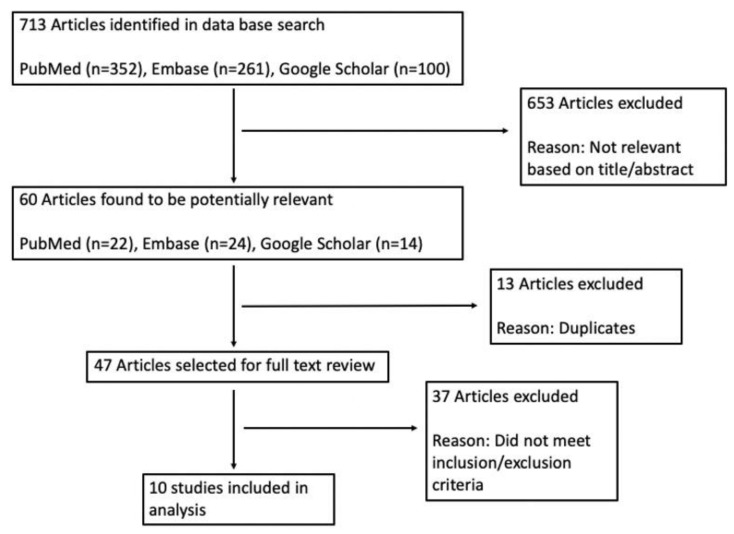
PRISMA diagram for literature review.

A total of 713 articles were initially identified for possible inclusion. Following a review of titles and abstracts, 653 articles were excluded. Of the remaining 60 articles, 13 were excluded as duplicates and 47 were assessed in full-text review. Finally, 37 articles were excluded for not meeting inclusion/exclusion criteria, leaving a final 10 studies to be included in this review (Figure 1).

Included studies focused on procedures in 13 different countries, with a plurality from the UK (4) and the US (3). Cataract surgery was by far the most represented ophthalmic subspecialty in the literature, with 8 of the 10 included studies exclusively discussing cataract surgery. Of the remaining two studies, one assessed trabeculectomy and the other focused on vitreoretinal surgery with gas tamponade (Table 1). Five of the studies reported both carbon footprint and solid-waste production, while three reported only carbon emissions and two reported solid waste.

A total of eight articles reported carbon emissions data (Supplementary Information). The raw numbers provided by each study were evaluated without checking the boundary conditions. The average emissions per surgery was 85.2 KgCO_2_eq, median 89 KgCO_2_eq (5.9–181.8). When separated by UN classification of developing vs. developed nations, developing countries have a lower net carbon footprint, at 73.1 KgCO_2_eq, compared to 100 KgCO_2_eq for developed nations. Additionally, the average carbon equivalents produced from phacoemulsification was 88.7 KgCO_2_eq, compared to 80.29 KgCO_2_eq for MSICS.

**Figure 2:**
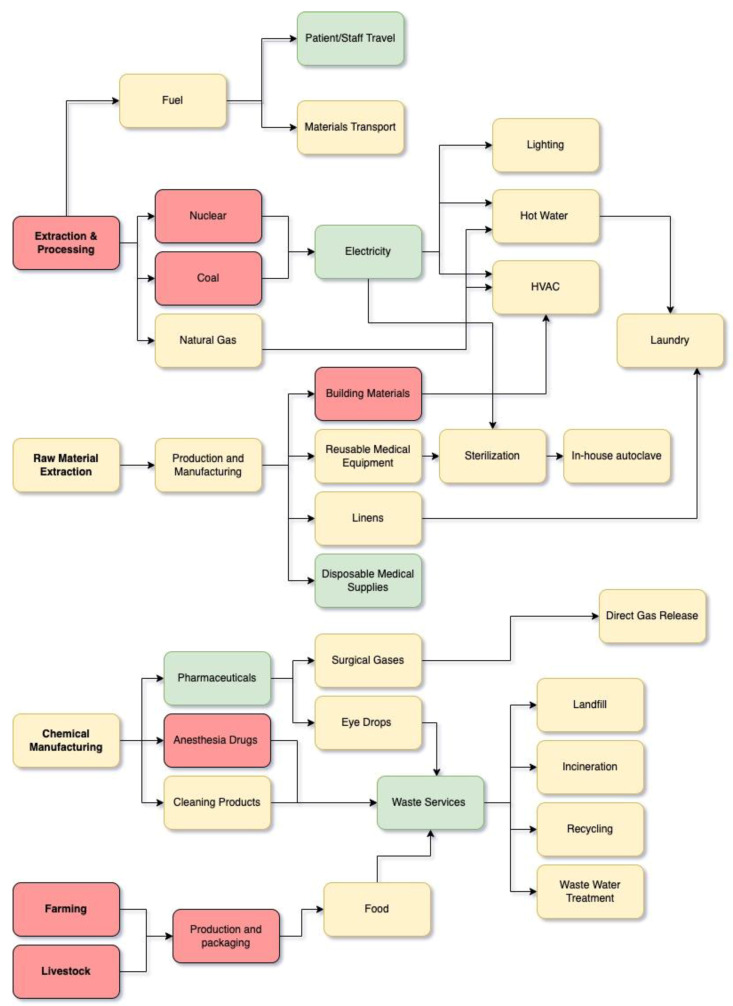
Overview of waste production lifecycle. Red depicts categories not represented in included articles, yellow indicates 1–4 articles and green indicates 5+ articles.

To quantify a net carbon footprint per surgery, each study had to establish scope and boundary conditions for which elements would be included in the estimate of carbon emissions for an individual surgery. After reviewing these articles, it is apparent that boundary conditions were set differently by different authors, making it difficult to compare the studies (Figure 2). For the purpose of this review, we will categorize carbon emissions into three categories: travel data (3a), direct building energy use (3b) and procurement data (3c).

### 3.1. Carbon Emissions—Travel

Of the eight studies that included carbon emissions data, five included information on emissions from patient or staff travel (Table 2). Data feeding into this category of emissions ranged from primary (collected via electronic records or questionnaires) to estimates of distances, modes of travel, and even numbers of staff required for steps in the ophthalmic process.

Somner et al. only included travel data from patients, and did not discuss staff travel. In contrast, Ferrero et al., Latta et al., and Morris et al. included staff travel in addition to patient travel data [16,17,22]. Ferrero assumed two surgeons, a resident, two nurses, and a healthcare assistant, who all performed 12 uncomplicated cataract surgeries in a single day [16]. The transport of the staff involved with sterilization of the phacoemulsification handpiece was also included in the calculations on carbon emissions.

Distance traveled and mode of travel (car, bus, etc.) affect the emissions estimates, and data for this were collected or estimated in different ways. Somner et al. calculated the mean distance of a round-trip journey to the clinic for 50 consecutive patients and assumed travel by car [23]. Morris et al. utilized a questionnaire including departure location and mode of travel [22]. Travel distances were calculated using Google Maps. Similarly, Ferrero et al. collected patients and staff addresses, as well as the mode of transport [16]. Distances were calculated using Google Maps and then converted to carbon emissions. Latta et al. and Goel et al. utilized the “Eyefficiency” application to record data for the calculation of carbon emissions [17,18]. Eyefficiency is a website and mobile phone application that was used to record specific data of the surgical procedures [18]. This information includes basic information on the surgical facilities, time and motion data during the surgery, and supply/pharmaceutical costs. Included in the list of information collected were travel data for patients and staff. Goel et al. do not specify exactly how they calculated travel data; however, they reported the same carbon emissions for both phacoemulsification and MSICS procedures [18]. Latta et al. collected travel methods from all staff in the operating theatre and the first 10 patients at each hospital location [17]. Everyone was assumed to have travelled by car, and the emissions were calculated using the average fuel emissions of the 2010 Toyota Corolla 1.6 L engine.

Some studies included travel for additional steps on the patient pathway, beyond travel for surgery itself. For example, Somner et al. used a “5-Stop” approach for phacoemulsification, including travel emissions from the following: first referral, preoperative assessment, clinic, surgery, first-day postoperative examination, and 1-month postoperative refraction [23]. In contrast, they defined MSICS as the “1-Stop” approach assuming a single visit on the day of the surgery. Due to the different number of appointments needed for each surgery type, the amount of travel emissions was expected to be different. Morris et al. assumed each patient travelled to the clinic for three appointments (initial assessment, surgery and post-operative follow up), the outpatient clinic required 5 staff members, the operating theatre utilized 12 staff members, and these staff members provided care for 12 patients [22].

### 3.2. Carbon Emissions—Building Energy

Six of the ten studies reported carbon emissions that can be attributed to the electricity or diesel used to perform the surgery (Table 2). Two studies report the exact amount of electricity (kWh) used to perform the procedures, while the remaining four only report the carbon emissions attributed to the electricity usage. Somner et al. only recorded the electricity usage from the phacoemulsification machine used during cataract surgery [23]. Since this was their only measure of energy, no carbon emissions were reported for MSICS in this category. Each phacoemulsification procedure was found to use 0.168 kWh of electricity, which correlated to approximately 0.078 kg CO_2_eq. They did not specific the exact conversion method used to calculate the carbon emissions. They calculated this electricity use as representing only 0.21% of the total carbon emissions for a single phacoemulsification surgery.

Morris et al. reported a more substantial contribution of energy usage to the total carbon production [22]. They calculated the total electricity usage during phacoemulsification by looking at two factors: the floor space required to perform the surgery and the total time that floor space was needed. In this study, the floor space includes both the operating room and the recovery areas. It was assumed that the energy usage of the operating theatre and recovery area was twice that of the mean energy used for the main hospital floor. They did not report the total amount of electricity (kWh) recorded. The conversion factor used was 0.59368 kg CO_2_eq per kWh. This study reported a total of 65.7 kg CO_2_eq, contributing 36.1% of the total emissions per surgery.

Goel et al., in 2021, employed similar strategies to calculate emissions from building energy [18]. As stated above, they utilized Eyefficiency to record detailed time and motion (TAM) data for each of their surgeries. The following time milestones were recorded for each surgery: patient on operating table, drape on the patient, first incision, incision closed, drape removed, patient off the operating table. Using these data, in combination with the operating room floor space and total energy usage of the hospital, electricity usage per procedure could be calculated. However, the intensity factor used to convert floor space to energy was not specified in this study. They excluded carbon emissions from non-electric heating sources, so the total emissions are likely to be underestimated in colder climates. As in Morris et. al, carbon emissions from electricity were found to be the same for both phacoemulsification and MSICS procedures [22]. The contribution of electricity to carbon emissions was found to range from 0.64 kg CO_2_eq (0.53%) in Mexico to 51.45 kg CO_2_eq (52.5%) in Swaziland for phacoemulsification. A similar distribution was found for MSICS. Goel et al. noted that the high proportion of total carbon emissions from electricity in Swaziland was likely due to the operating room only running 2 days a week and the assumptions made in the Eyefficiency application [18].

Latta et al. calculated energy consumption by acquiring the total monthly energy usage of each hospital or surgical unit involved in the study [17]. They then used the floor space and number of scheduled ophthalmology operating room days to calculate the energy used which was dedicated to the relevant surgeries. Like Morris et. al, they assumed that the surgical suite used twice the energy per floor space as the rest of the hospital [22]. They found that the surgical suite generated 17.8 kWh of electricity and 1.8 KgCO_2_eq. This was only 1.2% of the total carbon emissions for each case. Ferrero et al. utilized a similar strategy but also accounted for the operating room ventilation system and estimated the energy needed to sterilize the phacoemulsification handpiece [16]. It was noted that the sterilization of the handpiece was negligible, compared to the rest of the activities of the sterilization department. The carbon emissions reported were 0.75 KgCO_2_eq (0.76%) per case.

A more detailed approach was used by Thiel et al., to calculate emissions in this category at the Aravind Eye hospital in India [21]. They calculated electricity by obtaining the power ratings for all the equipment found in the operating room, including lighting. They assumed that each cataract surgery would take 9 min. They also included the energy for heating, ventilation, air conditioning (HVAC); sterilization of reusable equipment; laundry; and usage from burning diesel fuel in back-up generators. The exact numbers in carbon equivalents attributed to energy usage were not provided.

### 3.3. Carbon Emissions—Procurement

Six of the studies included procurement data in their carbon emissions estimates (Table 2 and Table 3). The studies use different specific features to define procurement, but it generally refers to the carbon emissions generated in the production and distribution of a particular product or products. It may include disposable supplies (custom packs, etc.), reusable supplies (instrument trays), linens, and even food. It may also include emissions from disposal of the product, and in the case of reusable items, their cleaning and sterilization between uses.

The first study to incorporate procurement into their analysis of carbon emissions was Morris et al., in 2013 [22]. The scope of their study included procurement for pharmaceuticals, disposable medical equipment, food, water, laundry and waste services. In their study, the Department of Environmental, Food and Rural Affairs (DEFRA) conversion factors were used to calculate the amount of carbon produced from the procurement of each of these products. From this study, it was shown that each phacoemulsification procedure produces 97.8 KgCO_2_eq, which is 53.8% of the total emissions for this procedure.

The next study to conduct a detailed look into procurement emissions was Thiel et al., at the Aravind Eye Care System [21]. This study had a similar scope to Morris et al., and included pharmaceuticals, disposable medical equipment, laundry, water, and waste services [22]. To calculate the carbon emissions for all listed items except for pharmaceuticals, they used a process-based LCA approach and the Ecoinvent database. For pharmaceutical products, they used an Economic Input Output Lifecycle Assessment (EIO-LCA), thus creating a hybrid LCA model. This method uses data on money spent within a particular economic sector, in this case pharmaceuticals, to generate a theoretical amount of associated carbon emissions. The exact amount of carbon emissions attributed to procurement was not quantified in this study, but based on figures included in their study, it represented the majority of the carbon emissions per surgery.

Tauber et al. also utilized EIO-LCA, as the scope of their study only included pharmaceuticals [20]. This study looked at the carbon emissions from pharmaceuticals at four different hospitals in the United States. The largest portion of pharmaceuticals left unused across all four sites were from eyedrops, followed by systemic medications and then injections. This trend was true for all sites except for the tertiary care center and the federal medical center. The reported carbon emissions from unused pharmaceuticals per month at each site were 418 kg CO_2_-e/month at the federal medical center, 711 kg CO_2_-e/month at the outpatient center, 2135 kg CO_2_-e/month at the ambulatory care center and 2498 kg CO_2_-e/month at the tertiary care center. In terms of carbon emissions per surgery, it was reported that this ranges from 6 to 30 KgCO_2_eq.

Goel et al. compared procurement emissions from phacoemulsification and MSICS [18]. The scope of procurement included reusable medical supplies, disposable medical supplies and waste services. The emissions were calculated using an LCA and EIO-LCA approach similar to that of Thiel et. al [21]. The carbon emissions from procurement were found to range from 9.76 KgCO_2_eq (9.96%) to 85.8 KgCO_2_eq (70.91%), with an average of 41.45 KgCO_2_eq (41.86%) for phacoemulsification. MSICS was found to range from 6.29 KgCO_2_eq (6.63%) to 58.81 (62.61%), with an average of 35.5 KgCO_2_eq (40.08%). For both surgery types, most waste came from disposable supplies, comprising 90% of the procurement emissions for phacoemulsification and 87.6% for MSICS.

Latta et al. included pharmaceuticals, medical equipment, waste emissions, and waste freight [17]. To calculate the carbon emissions for all these categories, they used the following conversion coefficients. They found that the total emissions per surgery from procurement was 127.2 KgCO_2_eq, which is 83.73% of the total emissions. From this percentage, 76.7% came from procurement of supplies, 6.9% came from procurement of pharmaceuticals and 0.13% came from disposal of the waste.

Ferrero et al. conducted a similar study to Latta et al., by including pharmaceuticals, medical equipment, and waste treatment services [16,17]. This study does not distinguish between reusable and disposable products. They utilized the EIO-LCA method to calculate carbon emissions for each of the substances included in scope of the study. For an uncomplicated cataract surgery, they found that procurement produced 75.23 KgCO_2_eq, which is 92.71% of the total emissions for each procedure. More specifically 73.32% can be attributed to procurement of medical devices, 12.68% for pharmaceuticals, 6.71% for the transport of these products and 1.39% for the waste services/disposal.

### 3.4. Carbon Emissions—Gas Tamponade (End of Life)

Moussa et al. was the only study to investigate carbon emissions from vitreoretinal surgery [25]. Specifically, they looked at the emissions from the gas tamponade procedure at three different medical centers in England. Each site utilized three different fluorinated gases, which are also greenhouse gases: SF6, C2F6, C3F8. In addition to this, each site used their own size of canister to perform the procedure. They measured the total amount of each gas used per procedure, and could then calculate the equivalents of carbon emissions based on the gases’ Global Warming Potential over 100 years (GWP100) [26]. Based on their calculations, they found that each procedure produced between 3.17 KgCO_2_eq when using a 30 mL canister and 124.8 KgCO_2_eq per patient when using a full cylinder (75 mL numbers not reported).

### 3.5. Solid Waste

A total of seven studies reported the solid waste produced from each procedure (Table 4). All studies give a reported breakdown of the waste except for Latta and Goel, who only report “total garbage” per case. The average mass of waste produced per cataract surgery was 990 grams (g), while the average for glaucoma surgeries was 767 g. When broken down by UN classification, cataract surgeries from developed nations produced an average of 1274 g, while cataract surgeries from developing nations produced 851 g, on average. Additionally, phacoemulsification produced more waste compared to MSICS, producing 1215 g and 465 g, respectively.

The first study that measured waste generated during any type of ophthalmologic surgery was Namburar et al., in 2008, where they compared the total waste generated per trabeculectomy at a US and an Indian hospital [24]. For 38 trabeculectomies, 44 trabeculectomies with phacoemulsification and 20 drainage device surgeries, they measured the waste generated in the three different waste streams at Aravind Eye Hospital (AEH) in India: infectious-waste landfill, infectious-waste incineration, and non-infectious/non-human waste. They did not specify what exactly was in each waste stream. In comparison, they only had data for five cases of trabeculectomies at the US community hospital. They found that AEH had significantly less waste generated per trabeculectomy (1400 ± 0.4 g vs. 500 g ± 200 g). There was not a significant difference in waste produced between trabeculectomy, trabeculectomy with phacoemulsification, and drainage device surgery at AEH.

Somner et al. (2009) only reported plastic and paper waste per cataract surgery, and compared the difference for this between phacoemulsification and MSICS [23]. They found that the MSICS produces a greater amount of plastic and paper waste (416 g) compared to phacoemulsification (128 g). Goel et al. also examined the amount of solid waste produced from phacoemulsification and MSICS [18]. However, Goel et al. did not break down the waste collected, but simply reported the “total garbage” as their solid waste [18].

Khor et al. (2020) and Thiel et al. (2017) investigated waste generated during cataract phacoemulsification surgeries [19,21]. Like Namburar et al. (2008), Thiel et al. (2017) conducted their study at AEH, finding that 250 g of waste is generated per phacoemulsification [21]. About 75% of the total waste in AEH is recycled. They further specified the mass of different material components in each waste stream (for example, specifying how much steel instruments contributed to single-use disposable waste). For simplicity, and so that results from their study are easily comparable to other studies that report waste, we report the totals of recycled, landfilled, and biohazardous waste (Table 4), without reporting the masses of every material recorded.

Khor et al. (2020) found that 814 g of waste was generated per phacoemulsification cataract surgery at their private Malaysian hospital, with about 15% of this being recycled [19]. They also report the material breakdown of their three different waste streams (recyclable waste, nonrecyclable waste, and clinical waste). To aid comparisons with the other studies, we adjusted their “clinical waste” mass so that it does not reflect the mass of liquids (povidone-iodine and balanced salt solutions) (Table 4).

## 4. Discussion

### 4.1. Carbon Emissions

When comparing emissions from different types of cataract surgeries, there is a noticeable difference in the carbon emissions produced from MSICS and phacoemulsification. This is well exemplified in Somner et al. in Scotland, who showed that phacoemulsification produces roughly five times more carbon emissions than MSICS per case [23]. However, Goel et al. demonstrated minimal difference in carbon equivalents produced [18]. This is likely caused by Somner et al.’s assumption that phacoemulsification requires a total of five visits and MSICS needs only one visit [23]. The energy used from the phacoemulsification machine is minor, making Somner’s data largely reliant on travel emissions. Goel, by contrast, assumed that emissions from travel were the same for both procedures and incorporated other sources of carbon emissions into their total calculation. The Eyefficiency tool also uses certain simplifying assumptions that make it more difficult to compare the CO_2_ differences between phacoemulsification and MSICs. These aspects limit the ability to compare MSICS and phacoemulsification procedures in this study. However, these findings exemplify the importance of considering travel when attempting to reduce carbon emissions.

To reduce carbon emissions from travel, we should reassess how many post-operative visits are needed without affecting surgical outcomes, and determine the optimal use of virtual care or telemedicine. One study investigating post-operative complications of cataract surgery showed that it was possible to refrain from post-operative visits altogether, without additional complications [27]. However, this was only the case when the surgery was uncomplicated and the patient did not have any medical comorbidities. Since a large portion of cataracts are simply age-related, this is an opportunity to cut down follow-up exams for healthy patients [28]. Additionally, the majority of post-operative appointments were patient-initiated, for visual disturbance, redness, pain or anxiety, most of which are common symptoms during normal recovery [29]. This also provides an opportunity to appropriately educate patients during pre-operative visits on the signs and symptoms that would warrant an additional in-person exam.

Carbon emissions also varied, based on geographic location. Morris et al. and Thiel et al. utilized a similar scope, but provided significantly different carbon emissions for surgeries performed in Wales and India, respectively [21,22]. In addition, Goel’s study analyzed emissions from many different countries, while using the same study design. India was shown to produce the least amount of emissions, while the most came from Hungary. This reinforces our finding that developing nations on average produce less carbon emissions compared to developed nations. Hence, analysis of the surgical process and procedures used in developed and developing nations may provide insight to further reduce carbon emissions. One unique example is the Aravind Eye Hospital (AEH) in India. This institution produces significantly lower emissions, due to the high volume of procedures performed per day, with the re-use and sterilization of surgical materials between cases [21]. This is in contrast to developed countries, who utilize many more single-use materials, mainly to reduce liability for complications such as infection [30]. However, examination of AEH shows that patients had comparable, if not better, outcomes compared to a facility in the United States [21].

It is important to note that accuracy in these geographic comparisons is limited by several factors. For instance, energy from heating sources was not included in the scope of Goel et. al [18]. This has the potential to underestimate the carbon emissions from cold climates, where energy used for heating is significantly higher. Additionally, the methodology of these studies requires providers to input data, which can contribute to human error. This can significantly affect accuracy, especially if there is a lack of training and standardization among providers. Finally, these studies used aggregate totals of supplies such as pharmaceuticals in their calculations, which may also reduce accuracy.

Procurement consistently contributes the largest percentage of total carbon emissions, followed by travel and building energy, respectively, in most studies. This is not surprising, since the surgical suite utilizes many supplies, many of which are single-use [31]. Several studies investigated which aspects of procurement contribute the most to total emissions. For instance, Thiel et al. report that the majority of procurement emissions are produced from medical supplies, followed by pharmaceuticals [21]. This also seems to be consistent with other studies that evaluate emissions from procurement. As mentioned above, AEH was shown to reduce medical supply usage by reusing phacoemulsification equipment between cases and rinsing gloves with an antiseptic solution. Tauber et al. further investigated the use of pharmaceuticals, finding that a large portion of unused pharmaceuticals comes from eyedrops [20]. AEH was also able to reduce emissions from pharmaceuticals by reusing medication bottles between cases. This is not the standard practice in many developed countries, due to perceived risk of medical complications, as well as billing practices and pharmaceutical dispensing laws. As an example, endophthalmitis is a serious complication of ophthalmologic surgeries, and can result in permanent vision loss [32]. However, these practices have not been shown to lead to an increased risk of post-operative infections such as endophthalmitis. Examination of AEH shows that patients had comparable, if not better, outcomes compared to a facility in the United States [21]. Due to this, we need to re-evaluate which precautions actually contribute to better outcomes for patients, and reduce waste from those that make little-to-no difference.

Although these findings do provide valuable insight, there are also several factors that limit the accuracy of these results. Procurement was the main focus of the majority of included studies, with many more contributing factors compared to that of building energy or travel. Because of this, building energy and travel are likely to be underestimated, relative to procurement. Additionally, the scope of procurement categories included varies significantly between each study. It would be expected that studies with a larger scope would produce higher carbon emission values than studies with a smaller scope. However, Latta and Ferrero utilize a similar scope and report procurement emission values that differ by 52 KgCO_2_eq per case. In addition, Morris utilized a broader scope than Latta, but reports lower emissions from procurement. This suggests that there are more factors that contribute to procurement emissions than the scope of the study alone, including methodological choices. As discussed above, location does seem to play a role, due to potential differences in heating requirements and operating room practices, as well as varying sources in energy mixes for electric grids. Another significant factor may be the strategy utilized to estimate carbon emissions from procurement. Among the three studies listed above, Morris uses LCA, Ferrero uses EIO-LCA and Latta uses an undefined method. Despite this lack of standardization among the included studies, these results do suggest that procurement of OR materials produces large amounts of carbon and that reducing this should be a priority.

Building energy was also shown to be a significant contributing factor in the production of carbon emissions. However, there was also significant variation between studies when calculating these emissions. For example, Somner et al. report values significantly lower than other studies [23]. This is because they only incorporate the energy used by the phacoemulsification machine. Morris et al. and Latta et al. utilized a similar strategy, based on OR floor space, but Morris et al. report values nearly 37 times higher [17,22]. This is because Morris et al. measured electricity from the entire cataract process, including pre- and post-operative visits, while Latta et al. only focused on the surgical procedure [17,22]. Another factor is the source of electricity utilized. It was noted that France produces fewer carbon emissions from electricity, due to the higher use of nuclear energy. Several studies also used Eyefficiency, which was likely able to provide the most accurate readings of energy use during the procedure, due to excluding energy during OR downtime. The combination of the large scope from Morris et al. and accurate recordings from Eyefficency would theoretically provide emission values that better reflect reality [22].

Only one study specifically looked at the carbon emissions from the use of gas tamponade in retinal procedures [25]. Although specific numbers were not provided for comparison, they showed that the use of 30 mL canisters instead of a cylinder can greatly reduce the GHG emissions from retinal procedures. The reported contribution from using a 30 mL canister was 3.17 kg CO_2_eq, which is low, compared to many cataract surgeries. However, this study did not include any other contributing factors to the carbon emissions such as travel, energy use, or procurement of other materials. Retina surgery, on average, takes significantly more time and uses more material than cataract surgery. This suggests that the total carbon emissions from retina surgery are likely to be much higher than from cataract surgery.

### 4.2. Solid Waste

Seven of the studies included in this review also included data on the amount of solid waste in cataract surgeries and trabeculectomy. Trends in solid-waste production closely follow those found in carbon emissions. For instance, more solid waste was produced in phacoemulsification compared to MSICS. Additionally, less waste was produced in developed countries compared to developing countries. This was also reflected in the results for glaucoma surgeries, where waste from a trabeculectomy in the USA was more than double that of the same procedure in India. The reasons for these trends are not fully understood, but they are likely to be similar to those mentioned above. Finally, the breakdown of solid-waste sources produces minimal information, due to the high degree of variability in how the waste was categorized. However, Somner et al. suggest that plastic contributes more to the total waste, compared to paper [23]. There does not seem to be a trend for infectious vs. non-infectious waste. More data are needed to understand the production of solid waste from ophthalmic surgeries.

### 4.3. Limitations

As discussed above, conclusions here are limited by the high degree of variability in the methods used in each of the included studies. Although these can provide general information about the sources of waste and relative contributions, the exact numbers are likely to vary substantially, based on location and practice patterns. In addition, this topic has been more extensively researched in recent years, and new, emerging studies are likely to utilize additional novel methods. Hence, this further exemplifies the need for standardization to draw stronger conclusions in this field.

## 5. Conclusions

GHG emissions have been shown to be highly variable among geographical regions, type of surgery, and individual practices of the participating facility. This variability provides an opportunity to optimize surgical and perioperative care practices, to reduce emissions from ophthalmologic surgeries. For instance, we can use evidence-based medicine to reduce the number of postoperative visits, when appropriate, which will not significantly alter visual outcomes. The reuse of materials in the OR can also significantly decrease procurement emissions without changing the risk of infection. Energy use in the OR can be reduced by increasing total patient throughput and reducing off-hour OR energy use. Even if a site has not performed a formal LCA, these principals can be applied immediately, to start reducing their carbon footprint.

More work is ultimately needed to understand sources of GHG emissions and asses the carbon footprint of other ocular subspecialties such as oculo-plastics, vitreoretinal, and strabismus. The comparability of studies should also be addressed as a greater level of standardization in future studies will allow for better meta-analysis. Studies should include a clearly defined scope and boundary conditions, clarify what is included or not included in the assessment, and utilize a standardized approach to LCA. Methods of assessing building energy are also heterogeneous, likely underestimating GHG emissions, and more should be done to help accurately estimate the energy consumption and GHGs in ORs globally. We hope that our suggested waste production lifecycle will help to standardize data collection methods, making studies comparable and making it easier to identify areas of improvement. These first steps not only provide an opportunity to increase the sustainability of ophthalmologic surgery, but to improve public health worldwide.

## Figures and Tables

**Table 1:** Study Characteristics. (Nations sorted by UN guidelines: A = Developed, B = Economies in transition, C = Developing countries).

Article	Study Type	Country	Subspecialty	# of Cases	Solid Waste	Carbon Emissions
Ferrero et al. [16]	Prospective Level 5	France (A)	Cataract	12	X	X
Latta et al. [17]	Prospective Level 3	New Zealand (A)	Cataract	142	X	X
Goel et al. [18]	Prospective Level 3	Global Facilities (A,C)	Cataract	475	X	X
Khor et al. [19]	Prospective Level 3/4	Malaysia (C)	Cataract	203	X	
Tauber et al. [20]	Prospective Level 3	USA (A)	Cataract	308/month		X
Thiel et al. [21]	Prospective Level 4	India (C)	Cataract	2942	X	X
Morris et al. [22]	Prospective Level 5	Wales/UK (A)	Cataract	1		X
Somner et al. [23]	Prospective Level 3	Scotland/UK (A)	Cataract	50	X	X
Namburar et al. [24]	Prospective Level 3	India (C), USA (A)	Glaucoma	AEH: 102 MAH: 5	X	
Moussa et al. [25]	Retrospective Level 3	England/UK (A)	Vitreoretinal	4877		X

**Table 2:** Carbon emissions attributed to travel, building energy and procurement.

Article	Location	Surgery	Travel (kg CO_2_)	Building Energy (kg CO_2_)	Procurement (kg CO_2_)	Total (kg CO_2_)
Somner et al. [23]	Scotland	Phaco	37.3 (98.2%)	0.078 (2.8%)	N/A	38
	Scotland	MSICS	7.5 (100%)	0 (0%)	N/A	7.5
Morris et al. [22]	Wales	Phaco	18.3 (10%)	65.7 (36.1%)	97.8 (53.8%)	181.8
Ferrero et al. [16]	France	Cataract	7.34 (9.0%)	0.75 (0.9%)	75.23 (92.7%)	81.13
Goel et al. [18]	Mexico 1	Phaco	73.2 (64.2%)	2.98 (2.6%)	38.82 (34.1%)	114
	Mexico 2	Phaco	34.4 (28.4%)	0.64 (0.5%)	85.8 (70.9%)	121
	Mexico	MSCIS	34.48 (36.7%)	0.64 (0.7%)	58.81 (62.6%)	93.93
	Chile	Phaco	62.3 (72.4%)	7.32 (9.6%)	16.18 (18.8%)	86
	Eswatini (Swaziland)	Phaco	37.16 (37.9%)	51.45 (52.5%)	9.76 (10%)	98
	Eswatini (Swaziland)	MSCIS	37.16 (39.4%)	51.45 (54.5%)	6.29 (6.7%)	94.4
	South Africa	Phaco	20.9 (38%)	13.33 (24.2%)	20.39 (37.1%)	55
	South Africa	MSCIS	20.9 (38.3%)	13.33 (24.4%)	20.39 (37.3%)	54.63
	India	Phaco	28.8 (70.2%)	1.43 (3.5%)	10.55 (25.7%)	41
	India	MSCIS	28.8 (71.2%)	1.43 (3.5%)	10.21 (25.2%)	40.44
	New Zealand	Phaco	32.62 (26.5%)	4.84 (3.9%)	85.29 (69.3%)	123
	New Zealand	MSCIS	32.62 (27.4%)	4.84 (4.1%)	81.8 (68.6%)	119.26
	UK	Phaco	19.2 (28.7%)	7.67 (11.4%)	40.29 (60.1%)	67
	Hungary	Phaco	50.1 (38.5%)	13.4 (10.3%)	66.57 (51.2%)	130
Latta et al. [17]	New Zealand	Phaco	22.8 (15%)	1.8 (1.2%)	127.2 (83.7%)	151.9
Tauber et al. [20]	USA	Phaco	N/A	N/A	6–30 (100%)	6–30
Thiel et al. [21]	India	Phaco	N/A	N/A	N/A	5.9
Average (KgCO_2_)			31.89	12.79	50.08	89.37
Percentage of total			35.68%	14.31%	56.04%	

**Table 3:** Scope of procurement and methods used to calculate procurement emissions.

Source	Ferrero	Latta	Goel	Tauber	Thiel	Morris
Pharmaceuticals	X	X	X	X	X	X
Reusable Medical Equipment	?	?	X			?
Disposable Supplies	X	X	X		X	X
Food						X
Laundry/Water					X	X
Waste Services	X	X	X		X	X
Methods						
Process-based LCA	?	?	X		X	X
EIO-LCA (financial)	X	?	X	X	X	

**Table 4:** List of solid waste produced per case reported in each study. (Nations sorted by UN guidelines: A = Developed, B = Economies in transition, C = Developing countries).

Study	Location	Procedure	Solid Waste per Case	Breakdown of the Waste
Namburar et al. (2008) [24]	India (C)	Trabeculectomy	500 g ± 200 g	Non-infectious/non-human waste: 300 g Infectious waste: 200 g
	India (C)	Tabeculectomy with Phaco	700 g ± 200 g	Non-infectious/non-human waste: 330 g Infectious waste: 150 g
	India (C)	Drainage Device Surgery	400 g ± 200 g	Non-infectious/non-human waste: 180 g Infectious waste: 210 g
	USA (A)	Trabeculectomy	1400 g ± 0.4 g	n/a
Somner et al. (2009) [23]	Scotland (A)	Phaco (5-Stop)	416 g	Plastic: 396 g Paper: 20 g
	Scotland (A)	MSICS (1-Stop)	128 g	Plastic: 116 g Paper: 12 g
Thiel et al. (2017) [21]	India (C)	Phaco	250 g	Recycled waste: 167 g Landfill/biomedical waste: 83 g
Khor et al. (2020) [19]	Malaysia (C)	Phaco	546 g	Noninfectious/Nonhazardous Waste: 311 g Nonrecyclable waste: 153 g Recyclable waste: 158 g Infectious/biohazardous Waste: 235 g
Ferrero et al. (2022) [16]	France (A)	Uncomplicated Cataract surgery	2830 ± 100 g	Unregulated Waste: 2660 g Hazardous Waste: 170 g
Latta et al. (2021) [17]	New Zealand (A)	Phaco	1320 g	Unspecified
Goel et al. (2021) [18]	Mexico 1 (C)	Phaco	670 g	Unspecified
	Mexico 2 (C)	Phaco	2230 g	
	Mexico (C)	MSCIS	2290 g	
	Chile (C)	Phaco	1320 g	
	Swaziland (C)	Phaco	190 g	
	Swaziland (C)	MSCIS	180 g	
	South Africa (C)	Phaco	1060 g	
	South Africa (C)	MSCIS	1060 g	
	India (C)	Phaco	870 g	
	India (C)	MSCIS	435 g	
	New Zealand (A)	Phaco	330 g	
	New Zealand (A)	MSCIS	190 g	
	UK (A)	Phaco	4270 g	
	Hungary (A)	Phaco	710 g	

## Data Availability

The datasets used and/or analyzed during the current study are available from the corresponding author upon reasonable request.

## Supplementary Materials

The following supporting information can be downloaded at: www.mdpi.com/article/10.3390/ijerph22010051/s1, Table S1: Search strategies.

## References

[1] Costello A., Abbas M., Allen A., Ball S., Bell S., Bellamy R., Friel S., Groce N., Johnson A., Kett M. (2009). Managing the health effects of climate change: Lancet and University College London Institute for Global Health Commission. Lancet.

[2] Clayton S. (2021). Climate Change and Mental Health. Curr. Environ. Health Rep..

[3] Ray C., Ming X. (2020). Climate Change and Human Health: A Review of Allergies, Autoimmunity and the Microbiome. Int. J. Environ. Res. Public Health.

[4] Ogden N.H. (2017). Climate change and vector-borne diseases of public health significance. FEMS Microbiol. Lett..

[5] Johnson G. (2005). The environment and the eye. Eye.

[6] Pachauri R.K., Meyer L., The Intergovernmental Panel on Climate Change (2015). Climate change 2014: Synthesis report. Contribution of Working Groups 1, 2, and 3 to the Fifth Assessment Report of the Intergovernmental Panel on Climate Change—IPCC.

[7] Richie C. (2022). Environmental sustainability and the carbon emissions of pharmaceuticals. J. Med. Ethics.

[8] MacNeill A.J., Lillywhite R., Brown C.J. (2017). The impact of surgery on global climate: A carbon footprinting study of operating theatres in three health systems. Lancet Planet Health.

[9] Rossi T., Romano M.R., Iannetta D., Romano V., Gualdi L., D’Agostino I., Ripandelli G. (2021). Cataract surgery practice patterns worldwide: A survey. BMJ Open Ophthalmol..

[10] Boulter T., Bernhisel A., Mamalis C., Zaugg B., Barlow W.R., Olson R.J., Pettey J.H. (2019). Phacoemulsification in review: Optimization of cataract removal in an in vitro setting. Surv. Ophthalmol..

[11] Shekhar M., Choudhury P., Ramya G., Sankarananthan R., Balagiri S., Wijesinghe H.K. (2022). Cataract surgery risk stratification in phacoemulsification and manual small incision cataract surgery in a teaching hospital. Int. Ophthalmol..

[12] Thiel C.L., Eckelman M., Guido R., Huddleston M., Landis A.E., Sherman J., Shrake S.O., Copley-Woods N., Bilec M.M. (2015). Environmental Impacts of Surgical Procedures: Life Cycle Assessment of Hysterectomy in the United States. Environ. Sci. Technol..

[13] Sherman J.D., MacNeill A., Thiel C. (2019). Reducing Pollution From the Health Care Industry. JAMA.

[14] Tricco A.C., Lillie E., Zarin W., O’Brien K.K., Colquhoun H., Levac D., Moher D., Peters M.D.J., Horsley T., Weeks L. (2018). PRISMA Extension for Scoping Reviews (PRISMA-ScR): Checklist and Explanation. Ann. Intern. Med..

[15] Oxman A.D. (1994). Systematic Reviews: Checklists for review articles. BMJ.

[16] Ferrero A., Thouvenin R., Hoogewoud F., Marcireau I., Offret O., Louison P., Monnet D., Brézin A.P. (2022). The carbon footprint of cataract surgery in a French University Hospital. J. Fr. Ophtalmol..

[17] Latta M., Shaw C., Gale J. (2021). The carbon footprint of cataract surgery in Wellington. N. Z. Med. J..

[18] Goel H., Wemyss T.A., Harris T., Steinbach I., Stancliffe R., Cassels-Brown A., Thomas P.B.M., Thiel C.L. (2021). Improving productivity, costs and environmental impact in International Eye Health Services: Using the ‘Eyefficiency’ cataract surgical services auditing tool to assess the value of cataract surgical services. BMJ Open Ophthalmol..

[19] Khor H.G., Cho I., Lee K.R.C.K., Chieng L.L. (2020). Waste production from phacoemulsification surgery. J. Cataract Refract. Surg..

[20] Tauber J., Chinwuba I., Kleyn D., Rothschild M., Kahn J., Thiel C.L. (2019). Quantification of the Cost and Potential Environmental Effects of Unused Pharmaceutical Products in Cataract Surgery. JAMA Ophthalmol..

[21] Thiel C.L., Schehlein E., Ravilla T., Ravindran R.D., Robin A.L., Saeedi O.J., Schuman J.S., Venkatesh R. (2017). Cataract surgery and environmental sustainability: Waste and lifecycle assessment of phacoemulsification at a private healthcare facility. J. Cataract. Refract. Surg..

[22] Morris D.S., Wright T., Somner J.E.A., Connor A. (2013). The carbon footprint of cataract surgery. Eye.

[23] Somner J., Scott K., Morris D., Gaskell A., Shepherd I. (2009). Ophthalmology carbon footprint: Something to be considered?. J. Cataract Refract. Surg..

[24] Namburar S., Pillai M., Varghese G., Thiel C., Robin A.L. (2018). Waste generated during glaucoma surgery: A comparison of two global facilities. Am. J. Ophthalmol. Case Rep..

[25] Moussa G., Ch’ng S.W., Park D.Y., Ziaei H., Jalil A., Patton N., Ivanova T., Lett K.S., Andreatta W. (2022). Environmental Effect of Fluorinated Gases in Vitreoretinal Surgery: A Multicenter Study of 4877 Patients. Am. J. Ophthalmol..

[26] AR6 Synthesis Report: Climate Change 2023—IPCC. https://www.ipcc.ch/report/sixth-assessment-report-cycle/.

[27] Westborg I., Mönestam E. (2017). Optimizing number of postoperative visits after cataract surgery: Safety perspective. J. Cataract. Refract. Surg..

[28] Thompson J., Lakhani N. (2015). Cataracts. Prim. Care.

[29] Porela-Tiihonen S., Kokki H., Kaarniranta K., Kokki M. (2016). Recovery after cataract surgery. Acta Ophthalmol..

[30] Hermer L.D., Brody H. (2010). Defensive Medicine, Cost Containment, and Reform. J. Gen. Intern. Med..

[31] Yeoh C.B., Lee K.J., Mathias S., Tollinche L.E. (2020). Challenges of Going Green in the Operating Room. Anaesth. Surg. Open Access J..

[32] Sheu S.J. (2017). Endophthalmitis. Korean J. Ophthalmol..

