# Supplementary material for: Waste and Greenhouse Gas Emissions Produced from Ophthalmic Surgeries: A Scoping Review
Source: Int J Environ Res Public Health. 2024 Dec 31;22(1):51. doi: 10.3390/ijerph22010051 (PMC11765210; doi:10.3390/ijerph22010051)
Supplement: Supplementary file 1 [file ijerph-22-00051-s001.zip › ijerph-3263284-supplementary.pdf]

Supplemental Table 1. Search strategies

Database	Search terms	Results
Pubmed	("Ophthalmology"[Mesh] or ophthalmology[text word] or opthalmology[title/abstract]) or ("operating room waste" or cataract or phacoemulsification[all fields] or cataract or "crystalline lens"[all fields]) and ("operating room" or "surgery"[all fields] or "surgical equipment"[all fields]) AND ((waste or "medical waste" OR emission* OR "carbon footprint" or footprint or carbon or greenhouse or recycling[title/abstract] or disposable[title/abstract])) AND (English[Filter])	339
Embase	('ophthalmology' OR 'eye disease') AND ('surgery' OR 'operating room' OR 'surgical equipment' OR 'cataract' OR 'phacoemulsification') AND ('waste' OR 'hospital waste' OR 'air pollution control' OR 'carbon footprint' OR 'greenhouse' OR 'greenhouse effect' OR 'recycling' OR 'disposable equipment')	117
Google Scholar	('ophthalmology' OR 'eye disease') AND ('surgery' OR 'operating room' OR 'surgical equipment' OR 'cataract' OR 'phacoemulsification') AND ('waste' OR 'hospital waste' OR 'air pollution control' OR 'carbon footprint' OR 'greenhouse' OR 'greenhouse effect' OR 'recycling' OR 'disposable equipment')	100
